# Ability of serum C-reactive protein and white blood cell cout in predicting acute schemic stroke. A short -term follow-up study

**Published:** 2016

**Authors:** Babak Bakhshayesh-Eghbali, Seyed-Ali Roudbary, Seddigheh Basir Jafari, Seyedeh-Parand Nabizadeh, Naghmeh Naderi-Asrami, Reza Sohrabnejad

**Affiliations:** 1 Department of Neurology, Guilan University of Medical Sciences, Rasht, Iran.; 2 Department of Neurology, Babol University of Medical Sciences, Babol, Iran.

**Keywords:** C reactive protein, White blood cell count, Ischemic Stroke, Prognosis. Outcome

## Abstract

**Background:**

Stroke is one of the leading causes of mortality and long-term morbidity. The aim of the present study was to determine the ability of baseline serum C-reactive protein (CRP) and white blood cell count (WBC) values in predicting the outcome of acute ischemic stroke (AIS).

**Methods:**

This study consisted of patients with first AIS referred to Poursina Hospital, Rasht, Iran. Severity of stroke was determined according to the National Institute of Health (NIH) Stroke Scale at the time of admission. Serum CRP levels and WBC count were measured at the time of admission. All patients were followed-up for 90 days after discharge and the severity of stroke was assessed using modified Rankin Scale. Receiver operating characteristic curve analysis was used for calculating the most appropriate cutoff point of CRP and WBC count for differentiating patients with and without poor outcome at the end of the study period.

**Results:**

A total of 53 out of 102 patients (52%) had poor outcome. The most appropriate cutoff value for CRP in differentiating patients with and without poor outcome was 8.5mg/l (sensitivity: 73.1%, specificity: 69.4%) and for WBC the difference did not reach to a significant level. The cutoff points of CRP > 10.5 mg/ml yielded a predictive ability at sensitivity: 75%, specificity: 63.8% whereas predictive ability of WBC for mortality was at a borderline level.

**Conclusion:**

These findings indicate that high levels of serum CRP in AIS at the time of admission is associated with poor prognosis. However, this study found no ability for WBC in predicting AIS outcome.

Stroke is one of the major health issues in developing countries and is one of the leading etiologies of mortality and long-term morbidity (1). Unfortunately, the crude number of people who suffer from stroke types annually, related deaths and disability- adjusted life years lost DALYs, is still increasing (2). Therefore, it is important to prevent acute ischemic stroke by determining and modifying risk factors. On the other hand, earlier initiation of effective reperfusion in patients with acute ischemic stroke is critical (3). So, the preventive strategies and treatment approaches for strokes are of particular importance. Over the past decade, many laboraty studies have found evidence of inflammation in the pathophysiology of cerebrovascular disease (4). Increase of several inflammatory cytokines, such as C-reactive protein (CRP), IL-6, IL1 have been found to have contribution in the pathogenesis of ischemic brain injury and worse neurological outcome (5, 6).

CRP, the most important inflammatory biomarker may play a role in progression of cerebrovascular pathologies (7, 8). There is conflicting evidence regarding the exact role of CRP as a prognostic biomarker in ischemic stroke outcome (9, 10). Similarly, the white blood cell count (WBC) has been also shown to predict the risk of first-time myocardial infarction and ischemic stroke (11, 12). 

It is well known that the prediction of outcome after ischemic stroke is important in clinical settings. However, identification of an independent prognostic marker in patients with stroke is still a matter of controversy. To our knowledge the data regarding predicting ability of serum CRP and WBC counts in patients with stroke are scarce. Thus, the aim of the present study was to determine the ability of serum CRP and WBC values assessed at the time of admission in predicting the outcome of acute ischemic stroke.

## Methods

***Patient selection and data collection:*** Patients with first-ever acute ischemic stroke who were referred to Poursina Hospital, Rasht, Iran in a one year period (2013-2014) were consecutively recruited in this cross-sectional study. The inclusion criteria were: onset of symptoms less than 24 hours and evidence of ischemic stroke on computed tomography (CT). Patients with history of previous cerebrovascular accidents (CVA), evidence of hemorrhagic stroke in CT, co-existing malignancy, end-stage liver or renal disease, active infectious diseases and use of anti-inflammatory agents were excluded from the study. 

Demographic data and clinical findings including ischemic heart disease (self-reported or use of cardiovascular drugs), hypertension (self-reported or use of anti-hypertensive agents), dyslipidemia (self-reported or use of anti-dyslipidemic agents), diabetes (self-reported or use of anti-diabetic agents) were recorded at the time of admission. Severity of stroke was determined by a neurologist using the National Institute of Health Stroke Scale (NIHSS) at the time of admission. 

The severity of stroke was categorized into three group based on NIHSS score (0-4 mild, 5-15 moderate and >16 severe). Serum inflammatory markers including WBC and CRP were measured at the admission time. CRP was measured quantitatively using BIONIK kit (Made in Iran, normal range: 4-6 milligram/liter).

***Follow-up:*** All patients were followed-up for 90 days after discharge from the hospital. The severity of stroke was assessed using modified Rankin scale (MRS). Patients with MRS score lower than 4 were considered as patients with good outcome and those with MRS score of 4 and higher as poor outcome patients. 

***Statistical Analysis:*** Statistical analysis was done using SPSS Version 16 (IBl cor, Chicago, USA). Kolmogrov-Smirnov test was used for testing normality in quantitative data. Chi-square and Fisher’s exact test were used for comparison of qualitative data. Multivariate regression logistic analysis was used to determine the predictors of poor outcome after 90 days of onset of stroke. Receiver operative characteristic (ROC) curve analysis was used for calculating the most appropriate cutoff point of CRP and WBC count to differentiate patients with and without poor outcome and mortality after 90 days. 

## Results

A total of 102 patients were recruited in this study. There were 43 (42.2%) males and 59 (57.8%) females and the mean age of patients was 69.471±12.125 years (range: 36-88). Table 1 shows the baseline characteristics of the patients. After 90 days of admission, 53 (52%) patients had poor outcome and 49 (48%) had good outcome. 

The cumulative rate of mortality was 32.4% (33/102). Median white blood cell count (8200±420.651 versus 9000±514.740 per micro liter, P=0.047) and CRP level (25±4.004 versus 5±1.837 milligram per liter, P<0.001) were significantly higher in patients with poor outcome compared to those with good outcome respectively. 

Multivariate logistic regression analysis was performed for controlling the potential confounding effect of age, chronic diseases, GCS and NIHSS at admission. CRP but not WBC count remained an independent predictor for poor outcome in patients with acute ischemic stroke (table 2).

**Figure 1:**
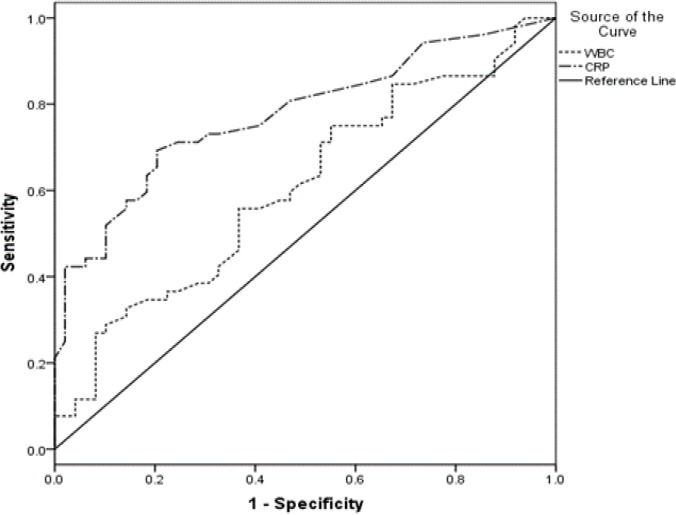
Receiver operative characteristic curve analysis for calculating the most appropriate cut-off point of CRP and WBC count for predicting poor outcome (AUC for CRP:0.777, 95%CI:0.686-0.867 and AUC for WBC count: 0.607, 95%CI:0.497-0.717

**Figure 2:**
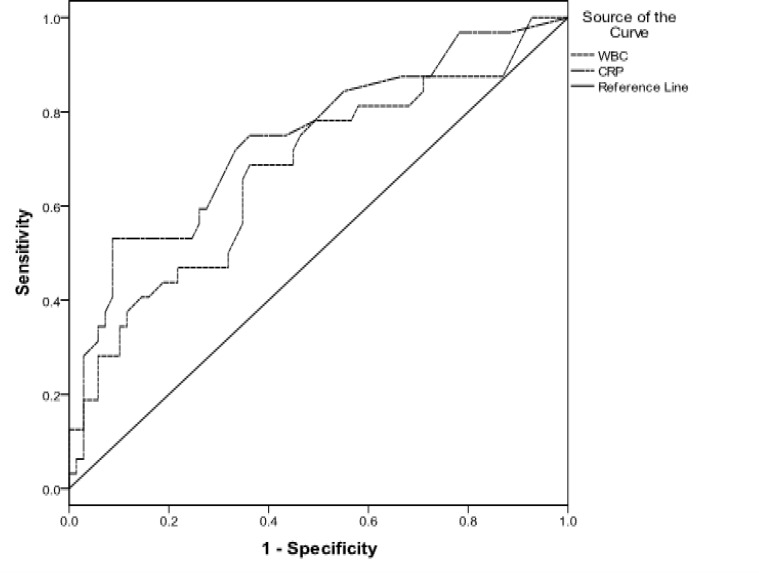
Receiver operative characteristic curve analysis for calculating the most appropriate cutoff point of CRP and WBC count for predicting mortality (AUC for CRP:0.743, 95%CI:0.636-0.850 and AUC for WBC count:0.675, 95%CI:0.558-0.792)

The most appropriate cutoff value for CRP in differentiating the two outcome groups was 8.5mg/l (sensitivity: 73.1%, specificity: 69.4%) and for WBC was 8.25×10^3^ per micro liter (sensitivity: 61.5%, specificity: 51%). However, the predictive ability of WBC did not reach to a significant level. Serum CRP > 10.5 mg/l predicted mortality at sensitivity of 75%, specificity of 63.8% whereas WBC did exhibit a borderline predictive ability for mortality (figures 1, 2).

**Table 1:** Baseline characteristics of patients (n=102)

**Variables**	**Good outcome (n=49)**	**Poor outcome (n=53)**	**Pvalue**
Age	65.388±11.800	73.245±11.261	0.001
Gender			
Male	23 (46.9)	20 (37.7)	0.347
Female	26 (53.1)	33 (62.3)	
Smoker	12 (24.5)	15 (28.3)	0.663
Chronic disease			
Ischemic heart disease	7 (14.3)	20 (37.7)	0.007
Diabetes	17 (34.7)	26 (49.1)	0.142
Hypertension	32 (65.3)	44 (83)	0.040
Dyslipidemia	17 (34.7)	28 (52.8)	0.065
GCS at admission			
15	46 (93.9)	29 (54.7)	<0.001
10-14	2 (4.1)	11 (20.8)	
<10	1 (2)	13 (24.5)	
NIHSS at admission			
Mild	31 (63.3)	16 (30.2)	0.003
Moderate	5 (10.2)	13 (24.5)	
Severe	13 (26.5)	24 (45.3)	
CRP	9.95±12.85	35.90±28.85	<0.001

GCS, Glasgow coma score. National Institute of Health Stroke Scale (NIHSS). CRP, C reactive protein

**Table 2:** Predictors for poor outcome in multiple logistic regression analysis (adjusted for chronic diseases

**Variable**	**Adjusted odd ratio**
WBC count at admission	1.043 (0.872-1.247)
CRP	1.041 (1.011-1.071)
Age	1.056 (1.009-1.106)
GCS<15 at admission	8.007 (1.796-35.704)
NIHSS at admission	
Mild	reference
Moderate	2.356 (0.739-7.516)
Severe	4.553 (1.074-19.298)

## Discussion

According to the findings of the current study, higher CRP levels not WBC on admission in acute ischemic stroke patients are associated with poor outcome. Ischemic brain injury begins a complex cascade which resulted in a systemic inflammatory response after both ischemic and hemorrhagic stroke (13). Different cytokines are involved in various aspects of stroke (14). Several studies have reported that higher levels of inflammatory markers such as CRP are associated with worse outcome after ischemic stroke (3, 15-16). According to pathophysiologic mechanisms of stroke, these findings may indicate different patterns of immuno-inflammatory activation (17). For example, a recent study indicated that CRP levels can predict the risk of recurrent strokes among lacunar stroke patients (18). It was documented that CRP level has a moderate prognostic factor to identify carotid stenosis (19). The results of this study are in agreement with previous studies (20-22) who demonstrated increased levels of serum CRP at admission was associated with worse outcome in patients with acute ischaemic stroke. There are data that suggest the post-stroke immune response occurs in a time-dependent period with the fact that the innate immune response occurring in the ﬁrst 24 hours following ischemic injury and theorized that the CRP is not sensitive enough for predicting beyond 24 hours and thus may not represent inflammatory status (23). Results from a population-based cohort in prediction of a 90-day subacute recurrent stroke revealed a weak significant association for C-reactive protein (24). We also report the appropriate cutoff of CRP for adverse consequences of stroke including poor outcome and mortality. There are limited studies calculating the appropriate cutoff of based on ROC curve analysis. Ghabaee et al. for the first time reported the cutoff of CRP value of 2.2 mg/l as the optimal cutoff value for the prediction of mortality within 7 days (sensitivity: 0.81, speciﬁcity: 0.80) (25). While in another similar study, CRP cutoff of 1.5 mg/dl was determined as the optimum sensitivity and speciﬁcity for adverse clinical outcome (26). Our study revealed higher amount of CRP as an appropriate cutoff point with an acceptable sensitivity and specificity. The difference between our measures and previous studies may be attributed to longer follow-up duration (e.g. 90 days). On the other hand, there are conflicting ideas against considering CRP as prognostic biomarker for ischemic stroke outcome. Because a large body of literatures are based on the studies conducting the relationship between CRP and ischemic stroke outcome by determination of mortality as an outcome measure. It is well-known that there are moderate to severe functional impairments in more than 50 percent of stroke patients (23). Therefore we tried to utilize the NIHSS score beside the mortality outcome.  Another factor that is suggested as a prognostic marker for outcome among patients with myocardial infarction and ischemic stroke is WBC count. Increasing the number of leukocytes could be a predisposing factor in high risk patients for ischemic stroke (27-28). The findings of the present study regarding predicting ability of WBC are in conrast with the study by Sahan et al. (29). Nerdi et al. have investigated the association of elevated WBC count at early stage (72 hours) of cerebral ischemia and found it a signiﬁcant independent predictor of poor clinical outcome, and discharge disability (30). Although blood biomarkers may provide valuable information regarding prediction of outcome in acute ischemic stroke but the ability of other acute phase reactant are different and this issue requires further prospective studies. In conclusion this study indicates that high serum CRP at the time of admission of acute stroke is predictive of poor outcome and serum levels greate than 10.5 mg/dl is predictive of mortality. 

## Notes

**Funding:** This study is the medical thesis of Seddigheh Basir Jafari**.**

## Citation:

Bakhshayesh-Eghbali B, Roudbary SA, Basir Jafari S, Ability of serum C-reactive protein and white blood cell cout in predicting acute schemic stroke. A short -term follow-up study. Caspian J Intern Med 2016; 7(3): 206-210.
